# Single-cycle influenza virus vaccine generates lung CD8^+^ Trm that cross-react against viral variants and subvert virus escape mutants

**DOI:** 10.1126/sciadv.adg3469

**Published:** 2023-09-08

**Authors:** Ming Z. M. Zheng, Tiong Kit Tan, Fernando Villalon-Letelier, Hilda Lau, Yi-Mo Deng, Svenja Fritzlar, Sophie A. Valkenburg, Haogao Gu, Leo L. M. Poon, Patrick C. Reading, Alain R. Townsend, Linda M. Wakim

**Affiliations:** ^1^ Department of Microbiology and Immunology, The University of Melbourne, Peter Doherty Institute for Infection and Immunity, Melbourne, Victoria 3000, Australia.; ^2^ MRC Human Immunology Unit, MRC Weatherall Institute of Molecular Medicine, University of Oxford, OX3 9DS Oxford, UK.; ^3^ WHO Collaborating Centre for Reference and Research on Influenza, Victorian Infectious Diseases Reference Laboratory, Peter Doherty Institute for Infection and Immunity, Melbourne, Victoria 3000, Australia.; ^4^ HKU-Pasteur Research Pole, School of Public Health, Li Ka Shing Faculty of Medicine, The University of Hong Kong, Hong Kong SAR, China.; ^5^ Division of Public Health Laboratory Sciences, School of Public Health, Li Ka Shing Faculty of Medicine, The University of Hong Kong, Hong Kong SAR, China.; ^6^ Centre for Immunology & Infection, Hong Kong Science Park, Hong Kong SAR, China.; ^7^ Centre for Translational Immunology, Chinese Academy of Medical Sciences, Oxford Institute, University of Oxford, OX3 7FZ Oxford, UK.

## Abstract

Influenza virus–specific tissue-resident memory (Trm) CD8^+^ T cells located along the respiratory tract provide cross-strain protection against a breadth of influenza viruses. We show that immunization with a single-cycle influenza virus vaccine candidate (S-FLU) results in the deposition of influenza virus nucleoprotein (NP)–specific CD8^+^ Trm along the respiratory tract that were more cross-reactive against viral variants and less likely to drive the development of cytotoxic T lymphocyte (CTL) escape mutants, as compared to the lung memory NP-specific CD8^+^ T cell pool established following influenza infection. This immune profile was linked to the limited inflammatory response evoked by S-FLU vaccination, which increased TCR repertoire diversity within the memory CD8^+^ T cell compartment. Cumulatively, this work shows that S-FLU vaccination evokes a clonally diverse, cross-reactive memory CD8^+^ T cell pool, which protects against severe disease without driving the virus to rapidly evolve and escape, and thus represents an attractive vaccine for use against rapidly mutating influenza viruses.

## Teaser

S-FLU vaccine protects against the flu and discourages the emergence of viral escape variants.

## INTRODUCTION

Influenza virus remains a major global health threat. It is a highly contagious, rapidly spreading severe acute respiratory pathogen with the potential to generate novel strains capable of global pandemics. While current influenza virus vaccines rely on the induction of neutralizing antibodies to strain-specific surface glycoproteins, this form of immunity leaves the population vulnerable to drifted seasonal or newly emerging pandemic strains. Developing vaccines that provide universal protection to circulating and emerging influenza virus strains remains a health issue of utmost global importance.

The induction of memory CD8^+^ T cell immunity is an effective means of inducing long-lived cross-protection against different influenza virus strains (*1*–*4*). As CD8^+^ T cell immunity targets internal viral proteins that are highly conserved across different influenza viruses, these T cells have the potential to protect against a range of influenza virus strains, including those with pandemic potential (*5*–*8*). A specific subset of memory CD8^+^ T cells, which are marked by the coexpression of CD103 and CD69 and reside in the upper and lower respiratory tract, termed tissue-resident memory (Trm) (*9*, *10*), mediate this influenza virus cross-strain immunity (*1*–*3*). Hence, T cell–based influenza virus vaccines designed to impart optimal cross-strain immunity should aim to evoke CD8^+^ Trm along the respiratory tract.

S-FLU is a novel, replication-deficient influenza virus vaccine candidate that is protective in mice, ferrets, and pigs (*11*–*14*). S-FLU lacks expression of hemagglutinin (HA) through suppression of the HA signal sequence and therefore is limited to a single cycle of replication (*11*). While S-FLU immunization does induce influenza virus–specific T cells (*11*–*13*), it remains unclear whether the truncated infection cycle of this vaccine affects the quality and localization of the cellular immune response. Using a mouse model, we compared the CD8^+^ T cell response following S-FLU vaccination to that evoked following influenza virus infection. We show that S-FLU immunization of mice results in the deposition of influenza virus–specific CD8^+^ Trm along the respiratory tract and these cells are protective against heterosubtypic infection. S-FLU vaccination generated lung CD8^+^ Trm with lower affinity compared to those generated following natural infection, a phenotype that coincided with increased T cell receptor (TCR) repertoire diversity within the S-FLU–generated lung CD8^+^ memory T cell pool. This diverse TCR repertoire of the S-FLU–generated memory T cell pool, which was partly driven by the limited inflammatory profile evoked by this vaccination regime, had a greater capacity to cross-react against viral variants and was less likely to drive the development of cytotoxic T lymphocyte (CTL) escape mutants. Our results show that the S-FLU vaccination platform can generate a clonally diverse cross-reactive lung CD8^+^ memory T cell pool that can protect against severe disease without driving the virus to rapidly evolve and escape vaccine-induced immunity.

## RESULTS

### Intranasal immunization with S-FLU generates nucleoprotein-specific CD8^+^ Trm in the lung

**Fig. 1.**
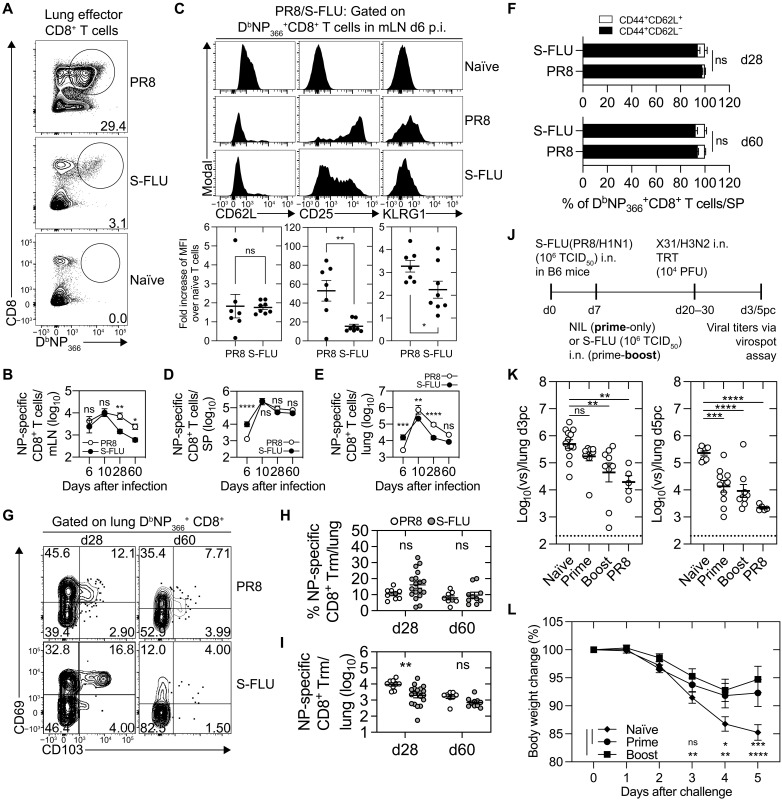
S-FLU immunization generates lung NP-specific CD8^+^ Trm that are protective against influenza challenge. B6 mice intranasally (i.n.) administered 10^6^ TCID_50_ S-FLU or 50 plaque-forming unit (PFU) of PR8 were analyzed for influenza virus–specific CD8^+^ T cells in the mLN, spleen (SP), and lung via flow cytometry. (**A**) Fluorescence-activated cell sorting (FACS) profiles of lung D^b^NP_366_^+^CD8^+^ T cells at day 10 after infection. (**B** to **E**) Absolute number of D^b^NP_366_^+^ CD8^+^ T cells in the (B) mLN, (D) spleen, and (E) lung. Data were pooled from two to eight experiments (*n* = 6 to 18 mice per group). Means ± SEM. Two-way analysis of variance (ANOVA) with Sidak’s multiple comparisons. (C) Expression of CD62L, CD25, and KLRG1 on mLN D^b^NP_366_^+^ CD8^+^ T cells at day 6 (d6) after infection. Symbols indicate individual mice. Means ± SEM. Two-tailed unpaired *t* test. (**F**) Frequency of splenic D^b^NP_366_^+^CD8^+^ T cells expressing CD44^+^CD62L^−^ or CD44^+^CD62L^+^ at days 28 to 30 after infection (top) and day 60 after infection (bottom). Data are pooled from two to three experiments (*n* = 8 to 10 mice per cohort). Means ± SEM. Two-way ANOVA with Sidak’s multiple comparisons. (**G**) Expression of CD69 and CD103 on lung D^b^NP_366_^+^CD8^+^ T cells at day 30/60 after infection with the corresponding (**H**) frequencies and (**I**) absolute cell numbers. Data are pooled from two to eight experiments (*n* = 6 to 18) mice per group. Means ± SEM. Symbols indicate individual mice. Two-way ANOVA with Sidak’s multiple comparisons. (**J**) B6 mice intranasally infected with 50 PFU of PR8/H1N1 or intranasally primed with 10^6^ TCID_50_ S-FLU(PR8/H1N1) and boosted 7 days later with S-FLU were rested for 20 to 40 days before an intranasal challenge with 10^4^ PFU of X-31/H3N2. (**K**) Viral titers in the lung on day 3 (left) and day 5 (right). Symbols represent individual mice. Means ± SEM. Pooled from five experiments, *n* = 5 to 13 mice per group. One-way ANOVA with Tukey’s multiple comparisons. (**L**) Weight loss, expressed as the percentage change of the original body weight following X-31 challenge. Data are pooled from four experiments (*n* = 9 to 10 mice per group). Means ± SEM. Two-way ANOVA with Tukey’s multiple comparisons. Two-way ANOVA with Tukey’s multiple comparisons test. *P < 0.05, **P < 0.01, ***P < 0.001, and ****P < 0.0001. ns, nonsignificant. p.i., post infection.

S-FLU is a replication-deficient influenza virus vaccine candidate. Because of suppression of the HA signal sequence, which prevents the synthesis of a viable HA glycoprotein, S-FLU is restricted to undergo only a single round of replication (*11*). To explore whether the truncated infection cycle of this S-FLU vaccine affects the size and localization of the influenza virus–specific CD8^+^ T cell response, we compared the kinetics of the CD8^+^ T cell response generated following S-FLU immunization to the response mounted following influenza virus infection. To this end, C57BL/6 mice were either vaccinated intranasally with 10^6^ median tissue culture infectious dose (TCID_50_) of S-FLU pseudotyped with PR8 H1 and N1 glycoproteins (S-FLU) or intranasally infected with influenza virus PR8 (A/Puerto Rico/8/34, H1N1). The influenza virus–specific CD8^+^ T cell response directed against the immunodominant epitope derived from the viral nucleoprotein (NP_366_) over the course of the infection was measured using an H-2D^b^ tetramer loaded with the NP_366_ peptide (Fig. 1A). At day 6 after infection/vaccination, equal numbers of NP-tetramer^+^ cells were present in the lung-draining mediastinal lymph node (mLN) of both cohorts (Fig. 1B); however, assessment of the expression of a panel of T cell activation markers revealed that NP-tetramer^+^ CD8^+^ T cells in the mLN of S-FLU–immunized mice expressed reduced levels of CD25 and Killer cell lectin-like receptor subfamily G member 1 (KLRG1) but equivalent levels of CD62L compared to NP-tetramer^+^ CD8^+^ T cells activated following PR8 infection (Fig. 1C). By day 10 after infection/vaccination, equivalent sized populations of NP-tetramer^+^ CD8^+^ T cells were observed in both the mLN and spleen of both cohorts; however, the number of NP-tetramer^+^ cells in the lung of influenza virus–infected animals now exceeded the numbers present in the respiratory tissue of the vaccinated cohort (Fig. 1, B, D, and E). At both early (day 28) and late (day 60) memory time points, the numbers of NP-specific CD8^+^ T cells in the mLN of S-FLU–vaccinated mice were significantly lower (4.8-fold) than the numbers present in influenza virus–infected mice, and while a similar trend was observed in the lung, the number and composition (predominately effector memory, CD44^+^CD62L^−^) of NP-tetramer^+^ CD8^+^ T cells in the spleen of vaccinated mice matched those generated following natural infection (Fig. 1F). Thus, S-FLU vaccination generates an effector CD8^+^ T cell pool similar to that generated following influenza virus infection. However, the size of the memory CD8^+^ T cell pools in the mLN and lung but not the spleen of the vaccinated cohort were reduced in comparison to those observed after natural infection.

Lung CD8^+^ Trm cells, which can be identified by the coexpression of the markers CD103 and CD69, are crucial in providing heterosubtypic protection against influenza virus (*1*–*4*). We next checked whether S-FLU vaccination resulted in the development of this tissue-bound memory T cell subset. While the frequency of NP-tetramer^+^ CD8^+^ T cells that converted into lung Trm, as assessed by the coexpression of CD103 and CD69 or via intravascular staining (fig. S1) at both early and late memory time points, was similar between the S-FLU–vaccinated and influenza virus–infected cohorts (Fig. 1, G and H), at early memory time points, there were 2.8-fold fewer NP-tetramer^+^ CD8^+^ Trm cells in the lung of S-FLU–vaccinated mice in comparison to the influenza virus–infected group (Fig. 1I). By day 60 after immunization, both S-FLU–vaccinated and influenza virus–infected mice had similar numbers of NP-tetramer^+^ CD8^+^ lung Trm, which correlated with an attenuated rate of decay of S-FLU–generated NP-tetramer^+^ CD8^+^ lung Trm (S-FLU Trm decayed at a rate of 100 cells/day, while influenza virus–generated Trm decayed at a rate of 300 cells/day).

To determine whether immunity established following S-FLU vaccination could protect mice against influenza challenge, naïve mice, mice infected with PR8 influenza virus, or mice intranasally immunized once (prime) or twice (prime+boost), 20 or 30 days earlier with S-FLU(H1N1), were challenged intranasally with X-31 (H3N2, HK/1968) influenza virus (Fig. 1J). Providing a secondary S-FLU boost increased the numbers of NP-tetramer^+^ CD8^+^ memory T cells in both the lung and spleen (fig. S2). As the challenge virus expresses different HA and neuraminidase proteins to those incorporated into the S-FLU vaccine, we can largely exclude the contribution of the humoral response toward the surface glycoproteins in protection. Viral titers in the lung were determined 3 and 5 days later. On day 3 after challenge, animals primed and then boosted with the S-FLU vaccine harbored 13.6-fold less virus in the lung compared to the naïve cohort (Fig. 1K). By day 5 after challenge, both groups of vaccinated animals (prime and prime+boost) contained similar levels of virus to animals previously infected with PR8 influenza virus and significantly lower viral loads (17-fold and 25-fold, respectively) in the lung compared to naïve mice that received a primary X-31 infection (Fig. 1K). Both groups of vaccinated mice displayed very similar weight loss curves, with all animals losing <10% of their original body weight, while the naïve cohort lost significantly more weight over the course of the experiments (~15%; Fig. 1L). These data shows that intranasal immunization with S-FLU generates a cellular immune response that confers protection against influenza virus challenge. This is in agreement with previous reports that show that S-FLU immunization can provide both homologous and heterosubtypic protection in both mice and ferrets (*14*).

### S-FLU–generated lung-resident memory NP-specific CD8^+^ T cells have lower TCR affinity and a more diverse TCR repertoire compared to memory CD8^+^ T cells generated following natural infection

**Fig. 2.**
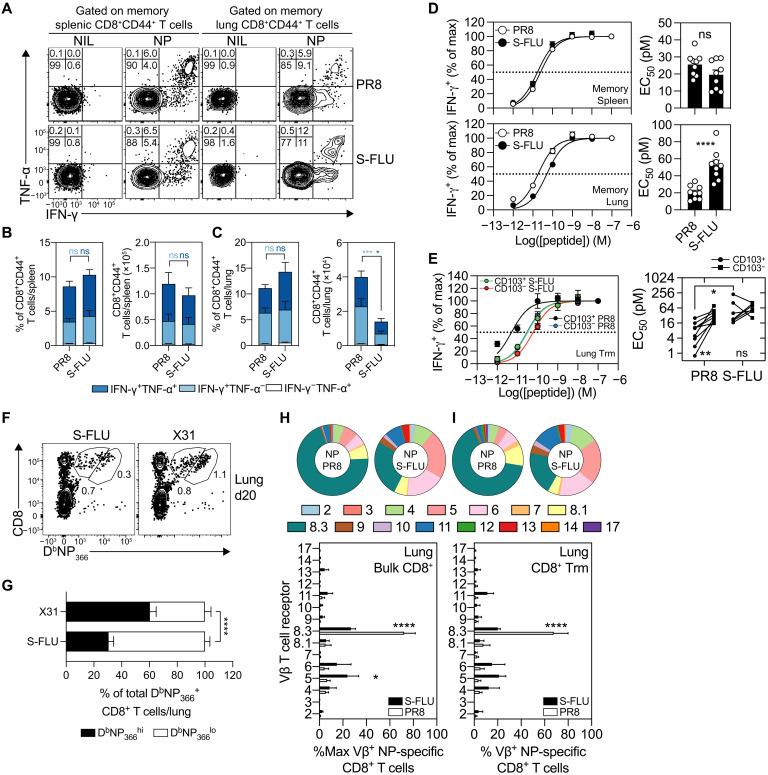
S-FLU–generated lung NP-specific CD8^+^ T cells have lower TCR affinity and a more diverse TCR repertoire compared to CD8^+^ T cells generated following natural infection. B6 mice intranasally administrated with 10^6^ TCID_50_ S-FLU or 50 PFU of PR8 and cytokine-producing CD8^+^ T cells at days 28 to 30 after infection in the spleen and lung were measured. (**A** to **C**) FACS panels of IFN-γ– and TNF-α–producing CD8^+^CD44^+^ T cells following NP_366_-peptide stimulation with corresponding frequencies (left) and absolute cell numbers (right) in the (B) spleen and (C) lung. Data are pooled from *n* = 6 mice from two experiments. Means ± SEM. Two-way ANOVA with Šidák’s multiple comparisons. (**D** and **E**) Peptide titration curves of the %max of IFN-γ^+^ production of (D) bulk and (E) CD103^+^ memory CD8^+^CD44^+^ T cells stimulated with graded concentrations of NP_366_ peptide with the corresponding EC_50_ values. The dotted line represents the 50% maximum response. Pooled from two experiments, *n* = 7 to 9 mice per group. Each symbol in EC_50_ plots represents unique biological replicates (bulk CD8^+^ T cells) or paired biological replicates (CD103^+^CD8^+^ T cells). Unpaired two-tailed Mann-Whitney test. Paired two-tailed *t* test comparing CD103^+^ and CD103^−^ subsets. (**F** and **G**) FACS profiles depicting lung D^b^NP_366_^lo^ and D^b^NP_366_^hi^ memory CD8^+^ T cells with corresponding (G) frequencies of B6 mice that received 10^6^ TCID_50_ S-FLU(X-31) or 10^4^ PFU of X-31 intranasally at day 20 after infection as measured by flow cytometry. Data are pooled from two experiments (*n* = 8 mice per cohort). Means ± SEM. Two-way ANOVA with Šidák’s multiple comparisons. (**H** and **I**) Normalized frequencies of Vβ TCR for bulk D^b^NP_366_^+^ CD8^+^ T cells in the (H) lung and (I) lung Trm compartment in B6 mice that received 25 to 50 PFU of PR8 or 10^6^ TCID_50_ S-FLU(PR8) intranasally at day 30 after infection. Data are pooled from three experiments [*n* = 3 samples (total of six mice pooled into three samples)]. Means ± SEM. Two-way ANOVA with Šidák’s multiple comparisons. *P < 0.05, **P < 0.01, ***P < 0.001, and ****P < 0.0001.

We next tested the quality (polyfunctionality and affinity) of the influenza virus–specific CD8^+^ T cell response generated following S-FLU vaccination. To this end, C57BL/6 mice were vaccinated intranasally with S-FLU(H1N1) or intranasally infected with influenza virus (PR8), and on days 28 to 30 after infection, the capacity of NP-specific CD8^+^ T cells in the spleen and lung tissue to synthesize interferon-γ (IFN-γ) and tumor necrosis factor–α (TNF-α) after a brief in vitro stimulation with NP peptide were measured (Fig. 2A). While the absolute numbers of cytokine-producing cells in the spleen were similar between the vaccinated and infected cohorts, there was a threefold reduction in the absolute number of cytokine-producing cells in the lung of the S-FLU–vaccinated group compared to the number detected in the lung of influenza virus–infected animals (Fig. 2, B and C), which is reflective of the presence of fewer total NP-specific memory CD8^+^ T cells persisting in the lung following S-FLU vaccination (see Fig. 1E). Nonetheless, the frequency of CD8^+^ T cells synthesizing IFN-γ, TNF-α, or both cytokines in the lung and spleen following S-FLU vaccination or influenza virus infection were similar with 6 to 10% of the total CD8^+^CD44^+^ T cells in both tissue compartments synthesizing both these cytokines after restimulation (Fig. 2, B and C). Thus, proportionately, memory NP-specific CD8^+^ T cells generated by S-FLU immunization and influenza virus infection are equally polyfunctional.

To determine whether the truncated infection cycle of S-FLU vaccine regulates the antigen sensitivity of memory CD8^+^ T cells, C57BL/6 mice were vaccinated intranasally with S-FLU(H1N1) or intranasally infected with influenza virus (PR8), and on days 28 to 30 after infection, we tested the antigen sensitivity of endogenous NP-specific CD8^+^ T cells in the spleen and lung by ex vivo stimulation with titrated doses of NP peptide followed by intracellular cytokine staining for the effector cytokine, IFN-γ. We observed that infection or S-FLU vaccination generated NP-specific CD8^+^ memory T cells in the circulation (spleen) with equivalent antigen sensitivity as determined by the percentage of cells producing IFN-γ (Fig. 2D, top). The antigen sensitivity of the total NP-specific CD8^+^ T cells in the lung of influenza virus–infected mice was 2.6-fold higher than NP-specific CD8^+^ T cells generated following S-FLU vaccination, as measured by the peptide concentration required to obtain 50% of maximum IFN-γ production [effective concentration 50 (EC_50_); Fig. 2D, bottom]. In addition, the NP-specific Trm generated following a single S-FLU immunization were also significantly less sensitive to antigen in comparison to the matched subset generated following PR8 infection (Fig. 2E). As an additional approximate measure of antigen affinity, we also determined the amount of tetramer binding on a per-cell basis and found that influenza virus–specific CD8^+^ T cells from the lung of X31-infected mice had higher tetramer binding than those recovered from S-FLU–immunized animals (Fig. 2, F and G). Thus, influenza virus infection resulted in the development of a memory CD8^+^ T cell pool in the lung with higher antigen sensitivity compared to those that form following S-FLU vaccination.

Next, we explored whether differences in antigen sensitivity of the lung NP-specific memory CD8^+^ T cells following infection and vaccination coincided with differences in diversity of the TCR repertoire. To do this, we profiled the TCR Vβ usage of NP-specific memory CD8^+^ T cells isolated from the lung of C57BL/6 mice 30 days following intranasal vaccination with S-FLU(H1N1) or intranasal infection with influenza virus (PR8). Consistent with prior reports (*15*, *16*), we find that following influenza virus infection, NP-tetramer^+^ CD8^+^ T cells showed preferential usage of the Vβ8.3 element (60 to 80%; Fig. 2, H and I). In contrast, assessment of the bulk and Trm NP-tetramer^+^ CD8^+^ T cells in the lung of S-FLU–vaccinated mice showed increased diversity and no preferential skewing of the repertoire toward Vβ8.3 usage (Fig. 2, H and I). Thus, S-FLU–generated lung memory NP-specific CD8^+^ T cells have lower antigen sensitivity and a more diverse TCR repertoire compared to memory CD8^+^ T cells generated following natural infection.

### The lower abundance, reduced affinity, and increased TCR repertoire diversity within the lung memory NP-specific CD8^+^ T cell pool after S-FLU immunization is not driven by vaccine antigen dose

**Fig. 3.**
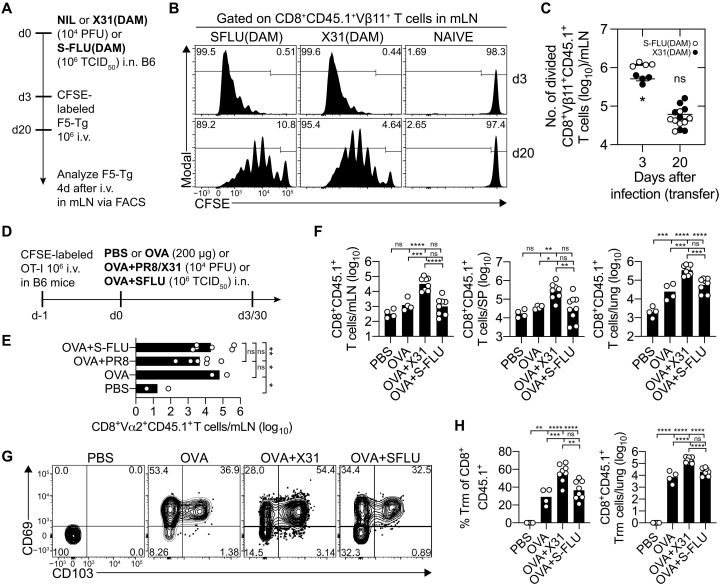
The lower abundance of lung memory NP-specific CD8^+^ T cell pool after S-FLU immunization is unrelated to antigen dose. (**A** to **C**) CD45.2 B6 mice intranasally administered with 10^4^ PFU of X-31-DAM or 10^6^ TCID_50_ X-31 S-FLU(DAM) were seeded with naïve CFSE-labeled CD45.1^+^CD8^+^ F5 T cells at days 3 and 20 after infection, and 4 days after transfer, the (B) frequency and (C) absolute cell numbers of divided F5 CD8^+^ T cells in the mLN were measured by flow cytometry. Data were pooled from one to two experiments (*n* = 4 to 7 mice). Means ± SEM. Symbols represent individual mice. Two-way ANOVA with Šidák’s multiple comparisons. (**D** to **H**) CD45.2 B6 mice were seeded with 10^6^ CD45.1 CFSE-labeled OT-I CD8^+^ T cells and, 1 day later, were intranasally administered either PBS, OVA (OVA, 200 μg), OVA and PR8/X-31 (10^4^ PFU), or OVA and S-FLU (10^6^ TCID_50_). At day 3/30 after infection, the number and phenotype of CD8^+^CD45.1^+^ OT-Is in the mLN, spleen, and lung were analyzed. (**E**) Absolute number of divided OT-I CD8^+^ T cells in the mLN at day 3 after infection. Data are pooled from two experiments, *n* = 2 to 6 mice. Bars indicate mean. Symbols represent individual mice. One-way ANOVA with Tukey’s multiple comparisons. (**F**) Absolute number of total CD8^+^CD45.1^+^ OT-I in the mLN (left), spleen (middle), and lung (right) at day 30 after infection. Data are pooled from three experiments (*n* = 4 to 8 mice). Bars indicate means. Symbols represent individual mice. One-way ANOVA with Tukey’s multiple comparisons. (**G** and **H**) FACS profiles of CD69^+^CD103^+^ CD8^+^CD45.1^+^ OT-I lung Trm with corresponding (H) frequencies (right) and numbers (left). Data are pooled from three experiments (*n* = 4 to 8 mice). Bars indicate means. Symbols represent individual mice. One-way ANOVA with Tukey’s multiple comparisons. *P < 0.05, **P < 0.01, ***P < 0.001, and ****P < 0.0001.

Differences in antigen availability and antigen load following influenza virus infection and S-FLU vaccination may be responsible for the differences in magnitude and affinity of the lung NP-specific memory CD8^+^ T cell response. Previous reports show evidence of prolonged presentation of viral antigen in the draining LNs of the respiratory tract following influenza virus infection and that this antigen retention controls the migratory pattern and activation state of virus-specific CD8^+^ T cells and boosts influenza virus–specific memory CD8^+^ T cell retention in the mLN (*17*, *18*). To check whether S-FLU vaccination results in the development of these residual antigen depots, C57BL/6 mice (CD45.2) were vaccinated intranasally with X-31 S-FLU(H3N1)-DAM [referred to hereafter as S-FLU(DAM)] or infected intranasally with X-31(DAM) virus; both viruses were engineered to express the NP epitope recognized by Clone F5 by site-directed mutagenesis of the NP. Hence, these viruses express the NP epitope recognized by CTL clone F5 NP(68)–specific CD8^+^ TCR transgenic cells (*19*, *20*). We then adoptively transferred 1 × 10^6^ naïve carboxyfluorescein succinimidyl ester (CFSE) labeled congenically marked (CD45.1^+^) F5 CD8^+^ T cells into mice at day 3 or day 20 after vaccination/infection. The proportion of divided transgenic F5.CD45.1^+^ CD8^+^ T cells in the mLN, measured as a loss of CFSE, was determined 4 days later (Fig. 3A). Transfer of F5 CD8^+^ T cells into mice 3 or 20 days after S-FLU(DAM) immunization or X-31(DAM) infection resulted in similar numbers of divided F5 CD8^+^ T cells being detected in the mLN (Fig. 3, B and C). Thus, like influenza virus infection, vaccination with S-FLU vaccine results in prolonged NP-antigen availability through the establishment of residual antigen depots.

We next tested whether differences in the magnitude of the lung memory CD8^+^ T cell response following influenza virus infection and S-FLU vaccination could be explained by differences in antigen load. To this end, we established a system using a model antigen, ovalbumin (OVA), and OT-I CD8^+^ TCR transgenic cells (specific for SIINFEKL epitope of OVA) to normalize the antigen dose between the infected and vaccinated cohorts. C57BL/6 mice (CD45.2) injected 1 day earlier with 10^6^ naïve CFSE-labeled OT-I.CD45.1^+^ CD8^+^ T cells were intranasally administered phosphate-buffered saline (PBS) or 200 μg of highly purified OVA protein either alone or in combination with influenza virus or S-FLU(H1N1), and 3 or 30 days later, OT-I.CD8^+^ T cell expansion and memory development were assessed (Fig. 3D). All cohorts that received OVA protein irrespective of whether it was coadministered with influenza virus or S-FLU had equivalent numbers of divided OT-I CD8^+^ T cells in the mLN at day 3 after treatment (Fig. 3E). However, by day 30 after treatment, there were clear differences in the memory OT-I CD8^+^ T cell compartment between these cohorts. Mice that received OVA protein and influenza virus had significantly more memory OT-I CD8^+^ T cells in the mLN, spleen, and lung compared to the cohort that was primed with OVA and S-FLU, which had a memory OT-I CD8^+^ T cell compartment in the profiled tissues similar in magnitude to that detected in animals that received OVA protein alone (Fig. 3F). Moreover, while 50% of OT-I CD8^+^ T cells in the lung of the OVA + influenza virus group adopted a Trm phenotype, this was reduced to 30% in the OVA+S-FLU cohort (Fig. 3, G and H). Collectively, the data shows that normalizing antigen availability and antigen load does not equilibrize the memory CD8^+^ T cell response that develops in the lung following S-FLU vaccination and influenza virus infection.

### The inflammatory profile evoked by S-FLU vaccination results in the reduced affinity and increased TCR repertoire diversity within the lung memory NP-specific CD8^+^ T cell pool

**Fig. 4.**
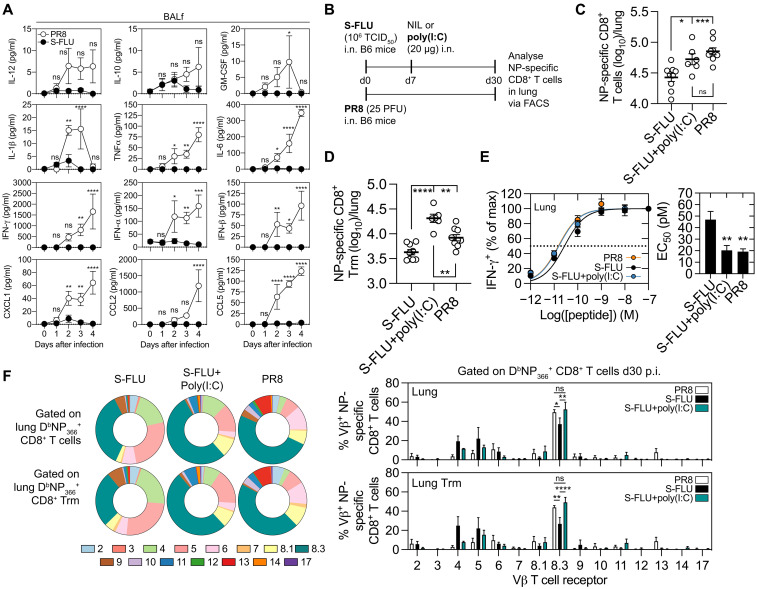
The lower abundance, reduced affinity, and increased TCR repertoire diversity within the lung memory NP-specific CD8^+^ T cell pool after S-FLU immunization is linked to the limited inflammatory profile evoked by the vaccination regime. (**A**) Cytokines and chemokines concentrations in the BALF of B6 mice intranasally immunized with 10^6^ TCID_50_ S-FLU or infected with 10^4^ PFU PR8 at days 1 to 4 after infection as measured by cytometric bead array. Data are pooled from three experiments (*n* = 4 to 6 mice per time point per cohort). Means ± SEM. Two-way ANOVA with Šidák’s multiple comparisons. (**B** to **F**) B6 mice were primed with 10^6^ TCID_50_ S-FLU or primed with S-FLU and boosted 7 days later with 20 μg of poly(I:C) [S-FLU + poly(I:C)], or mice were intranasally infected with 25 PFU of PR8, and 30 days later, the (C) total D^b^NP_366_^+^CD8^+^ T cells and (D) CD69^+^CD103^+^ D^b^NP_366_^+^CD8^+^ Trm in the lung were measured. Symbols represent individual mice. Data are pooled from three to four experiments (*n* = 6 to 9 mice per group). Means ± SEM. One-way ANOVA with Tukey’s multiple comparisons. (**E**) Peptide titration curves of the %max of IFN-γ^+^ CD8^+^CD44^+^ T cells in the lung that were stimulated with titrated concentrations of NP_366_ peptide with the corresponding EC_50_ values. Dotted line represents the 50% maximum response. Bars represent the mean of *n* = 4 to 12 mice per group from two to five experiments. Data are additionally pooled from (Fig. 2). Means ± SEM. One-way ANOVA with Dunnett’s multiple comparisons comparing the mean of each cohort against the baseline NIL cohort. (F) Normalized frequencies of individual Vβ TCR of total (top) and CD69^+^CD103^+^ Trm (bottom) D^b^NP_366_^+^CD8^+^ T cells in the lung. Means ± SEM. Data are from three experiments (*n* = 3 samples). Two-way ANOVA with Tukey’s multiple comparisons.*P < 0.05, **P < 0.01, ***P < 0.001, and ****P < 0.0001.

Inflammation is known to affect the magnitude and antigen sensitivity of the memory CD8^+^ T cell response (*21*, *22*). We next explored whether differences in the inflammatory milieu evoked following infection and S-FLU vaccination could explain the observed differences in the NP-specific lung memory CD8^+^ T cell compartment. To this end, C57BL/6 mice were either vaccinated intranasally with S-FLU(H1N1) or intranasally infected with influenza virus (PR8), and the inflammatory response in the lung were assessed by measuring the levels of a panel of cytokines/chemokines in the bronchial alveolar lavage fluid (BALF). By day 2 after infection, significant elevations in the levels of interleukin-1β (IL-1β), TNF-α, IL-6, IFN-γ, IFNα, IFNβ, CXCL1, CCL2, and CCL5 were detected in the airways of influenza virus–infected mice (Fig. 4A). In contrast, the amount of all inflammatory cytokines assessed in the airways of S-FLU–vaccinated mice failed to increase above baseline levels present in the airways of naïve control animals (Fig. 4A). The negligible levels of inflammatory cytokines and chemokines in airways following S-FLU vaccination also resulted in minimal innate immune cell (monocytes and neutrophils) recruitment into the airways (fig. S3) and reduced damage to the lung tissue compared to what was observed following influenza virus infection (fig. S4). Thus, in comparison to influenza virus infection, S-FLU vaccination evokes limited local inflammation and pulmonary damage.

We next explored whether reintroducing inflammatory cues during S-FLU vaccination via the coadministration of an adjuvant [poly(I:C)] would increase the magnitude and antigen sensitivity of the vaccine-induced NP-specific CD8^+^ T cell response. To this end, C57BL/6 mice were either intranasally infected with influenza virus (PR8) or vaccinated intranasally with S-FLU(H1N1) alone or in combination with poly(I:C), administered into the lung on day 7 after vaccination, which served to boost local inflammation in the lung (fig. S5). The size of the memory NP-specific CD8^+^ T cell response in the lung and the proportion that converted into Trm, in addition to the affinity and Vβ-usage, were measured 30 days later (Fig. 4B). The immunization regime of S-FLU and poly(I:C) boosted the absolute number of NP-specific CD8^+^ memory T cells in the lung twofold above that observed in animals given S-FLU alone, to a level similar to that observed in animals infected with influenza virus (Fig. 4C). Moreover, we observed a statistically significant increase in the number of NP-specific CD8^+^ Trm following S-FLU and poly(I:C) immunization (Fig. 4D). The antigen sensitivity of both NP-specific CD8^+^ T cells in the lung of influenza virus–infected mice and S-FLU– and poly(I:C)-vaccinated mice were equivalent and 2.3-fold higher than NP-specific CD8^+^ T cells generated following S-FLU vaccination alone (Fig. 4E). This increased antigen sensitivity of the lung NP-specific CD8^+^ T cells in the S-FLU– and poly(I:C)-vaccinated cohort coincided with an increased skewing of the TCR repertoire and greater Vβ8.3 usage, with the overall TCR Vβ repertoire following S-FLU and poly(I:C) immunization now mirroring that generated following influenza virus infection (Fig. 4F).

To confirm that the adjuvant, and not differences in antigen dose and availability, was narrowing the TCR repertoire, we administered intranasally highly purified OVA protein and S-FLU(H1N1) alone or in combination with poly(I:C) (as described above) and once again observed increased skewing of the OVA-specific CD8^+^ TCR repertoire following co-delivery of the adjuvant (fig. S6). This supports earlier reports that show a link between adjuvant and the clonotypic diversity of the T cell response (*23*). Thus, reintroducing inflammatory cues during S-FLU vaccination increases the magnitude and antigen sensitivity of the vaccine-induced lung NP-specific memory CD8^+^ T cell response, and this correlates with increased skewing of the TCR repertoire.

### S-FLU–generated lung memory NP-specific CD8^+^ T cells are cross-reactive and subvert the development of influenza virus CTL escape variants

**Fig. 5.**
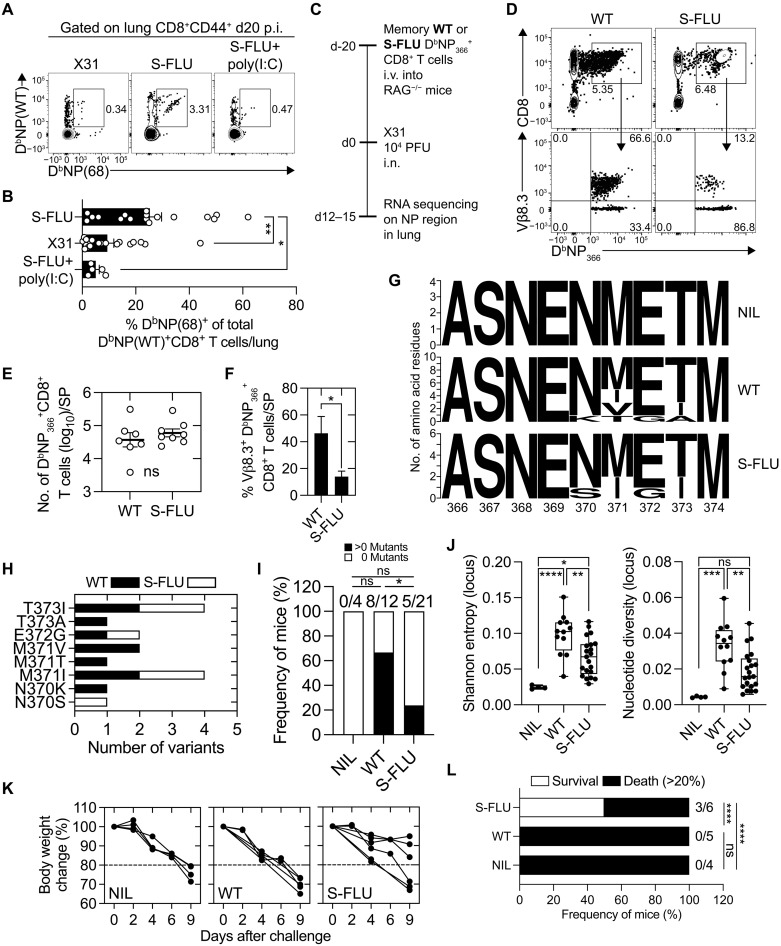
S-FLU–generated lung memory NP-specific CD8^+^ T cells subvert the development of influenza virus escape variants. (**A**) FACS profiles depicting frequency of lung D^b^NP(WT)^+^D^b^NP(68)^+^ CD8^+^CD44^+^ T cells at days 20 to 28 after infection of B6 mice intranasally immunized with 10^6^ TCID_50_ S-FLU(X-31), with a cohort additionally treated with 20 μg of poly(I:C) intranasally at day 7 after infection or infected with 10^4^ PFU X-31. (**B**) Frequency of D^b^NP(68)^+^ tetramer^+^ cells of the total D^b^NP(WT)^+^CD8^+^CD44^+^ T cells in lungs of mice described in (A). Data are pooled from five experiments (*n* = 5 to 21 mice). Means ± SEM. One-way ANOVA with Tukey’s multiple comparisons. (**C**) Memory D^b^NP_366-374_^+^ CD8^+^ T cells from lungs of wild-type (WT) influenza virus or S-FLU–administered B6 mice were intravenously (i.v.) transferred into RAG^−/−^ mice, which were rested, and then infected intranasally with 10^4^ PFU of X-31. At days 12 to 15, lung viral RNA was extracted and sequenced. (**D**) FACS profiles depicting Vß8.3 TCR expression on WT and S-FLU D^b^NP_366_^+^ CD8^+^ T cells in the lungs of donor mice. (**E**) The number of splenic D^b^NP_366_^+^ CD8^+^ T cells and the (**F**) Vß8.3 TCR frequency in RAG^−/−^ mice at days 12 or 15 after infection. Data are pooled from two experiments (*n* = 4 to 8 mice per group). Means ± SEM. Two-tailed unpaired *t* test. (**G** to **H**) Number of amino acid changes detected in WT and S-FLU RAG^−/−^ recipient cohorts depicted via (**G**) sequence logos or (**H**) bar plots. (**I**) Frequency of mice detected with at least one viral mutant. Two-sided Fisher’s exact test. (**J**) Shannon entropy and nucleotide diversity analysis on the viral population. Box plot lines display median and the first/third quartile. One-way ANOVA with Tukey’s multiple comparisons. Data are pooled from four experiments (*n* = 4 to 21 mice per group). (**K**) Weight change and (**L**) survival analysis (≥20% weight loss, dashed line) following X-31 challenge in the RAG^−/−^ adoptive transfer model. Data are pooled from two experiments (*n* = 4 to 6 mice per cohort). Two-sided Fisher’s exact test. *P < 0.05, **P < 0.01, ***P < 0.001, and ****P < 0.0001.

TCR diversity within a memory T cell pool is advantageous, as it can protect against, and limit the emergence of, viral CTL escape variants. We next tested whether the broad TCR repertoire diversity within the lung NP-specific CD8^+^ memory T cell compartment generated following S-FLU vaccination can cross-react with naturally occurring influenza viral variants and minimize T cell–mediated antigenic drift. To this end, C57BL/6 mice were intranasally infected with influenza virus X-31(H3N2), or vaccinated intranasally with S-FLU(H3N2), and on day 30 after infection, the proportion of cross-reactive NP-specific CD8^+^ memory T cells in the lungs were measured by simultaneous staining with two H-2D^b^ tetramers loaded with the NP_366_ peptide derived from either the parental/vaccine strain [ASNENMETM; NP(WT)] or a naturally occurring variant [ASNENMDAM; NP(68)], which differ in the sequence of the immunodominant NP CTL epitope at positions 7 and 8. Following S-FLU immunization, 20% of NP(WT)-specific CD8^+^ memory T cells in the lung cross-reacted with the variant NP(68) (ASNENMDAM) peptide, which was double that observed following influenza virus infection (Fig. 5, A and B). Both tetramers were highly specific and showed negligible levels of background staining in naïve mice (fig. S7). The extent of binding of the two major histocompatibility complex (MHC) tetramers was independent of the order in which the epitopes were encountered, as when we repeated the above experiment using the S-FLU(DAM) and X-31(DAM) as the primary strains, we once again observed significantly more cross-reactive NP(WT) CD8^+^ T cells in the lung of S-FLU(DAM)–vaccinated animals (fig. S8). The reintroduction of inflammation into the airways of S-FLU immunized mice by the administration of poly(I:C), following the immunization regime described above (see Fig. 4B) reduced NP-specific CD8^+^ T cell cross-reactivity (Fig. 5B), which is in line with our observation that this treatment increased skewing of the NP-specific TCR repertoire (Fig. 4F).Collectively, these data show that, in comparison to influenza virus infection, S-FLU vaccination evokes more influenza virus–reactive memory T cells that can cross-react against viral variants.

We next explored whether the broad TCR repertoire diversity within the lung NP-specific CD8^+^ memory T cell compartment generated following S-FLU vaccination can minimize antigenic drift. To do this in a controlled setting where we could exclusively assess the capacity of NP-specific CD8^+^ T cells to drive the emergence of viral escape mutants, we seeded RAG^−/−^ mice (lacks T and B cells) with NP-tetramer^+^ CD8^+^ memory T cells sort purified from the lungs of mice that were either infected with influenza virus or vaccinated with S-FLU(H1N1). We challenged these animals, and as a control, a cohort of RAG^−/−^ mice that did not receive a T cell transfer, intranasally with influenza virus, and then recovered the lungs and spleens on days 9 to 15 after infection (Fig. 5C). Assessment of the number of NP-tetramer^+^ CD8^+^ T cells in the spleen and lung shows that mice that received NP-tetramer^+^ CD8^+^ T cells from either S-FLU–vaccinated or influenza virus–infected donors (Fig. 5D) expanded to equivalent sized effector populations (Fig. 5E) and both T cell populations infiltrated the lung tissue with similar kinetics (fig. S9). Moreover, following infection, the NP-specific CD8^+^ T cells purified from influenza virus–infected donor mice retained their preferential usage of Vβ8.3 TCR, while NP-specific CD8^+^ T cells that originated from S-FLU–vaccinated donors retained their Vβ diversity (Fig. 5F and fig. S9).

Viral RNA was extracted from lung tissues, and the NP regions were sequenced to identify the emergence of NP-variants. In addition, we sequenced the input virus and confirmed the clonality of the WT-NP_366_ sequence. Viral RNA recovered from the lungs of RAG^−/−^ mice that did not receive any T cell transfer was clonal, with 100% of mice containing virus that expressed the WT-NP_366_ sequence (Fig. 5, G to I). Assessment of the viral RNA extracted from the lungs of RAG^−/−^ mice that received NP-tetramer^+^ CD8^+^ T cells from influenza virus–infected donor mice revealed the presence of mutations exclusively within the NP_366–374_ CTL epitope in 67% of animals (Fig. 5, G to I). These mutations were at positions 370 (N370K), 371 (M371➔I, M371➔V, M371➔T), 372 (E372➔G), and 373 (T373➔A, T373➔I), and most of these mutations have been previously reported to affect NP-specific CD8^+^ T cell recognition (*24*–*28*). In contrast, only 24% of animals that received NP-tetramer^+^ CD8^+^ T cells from S-FLU–vaccinated donor mice contained mutations within the NP_366–374_ CTL epitope (Fig. 5, G to I). Again, these mutations were concentrated at position 370 (N370➔S), 371 (M371➔I), 372 (E372➔G), and 373 (T373➔I). Analysis of the nucleotide diversity at the haplotype and locus level (estimated by nucleotide diversity and Shannon entropy) of the dataset showed statistically significant increase in epitope diversity in recipients of NP-specific CD8^+^ T cells derived from influenza virus infection compared to recipients of NP-specific CD8^+^ T cells derived from S-FLU immunization (Fig. 5J and fig. S10).

Assessment of the weight loss curves of these animals revealed that 100% of mice that received influenza virus infection–generated NP-specific CD8^+^ T cells lost ≥20% of their body weight (and were deemed moribund), which contrasts a 50% survival rate (<20% weight loss) in animals that were recipients of S-FLU–generated NP-specific CD8^+^ T cells (Fig. 5, K and L). These data may imply that increased TCR repertoire diversity within the NP-specific memory CD8^+^ T cell population generated following S-FLU vaccination applies less focused immune pressure, subverts the development of CTL escape variants, and reduces disease severity.

## DISCUSSION

Vaccination is the most reliable strategy to prevent influenza disease. Inactivated or recombinant seasonal influenza virus vaccines currently licensed for use rely heavily on the induction of humoral strain–specific responses to provide protection. This approach leaves the population vulnerable to antigenically drifted and antigenically shifted strains. Developing vaccines that provide universal protection to both circulating and emerging influenza virus strains remains an important health issue. The influenza virus–specific CD8^+^ T cell response is largely directed toward internal viral proteins that are highly conserved across different influenza viruses; hence, this type of immunity has the scope to induce long-lived cross-protection against different influenza virus strains (*6*–*8*). While these internal virus proteins are more conserved than viral surface proteins, T cell immunity is not infallible, and there is a risk that immune pressure can drive mutations within T cell epitopes, driving the emergence of quasispecies that can escape T cell immune control (*28*). Here, we find that a single-cycle influenza virus vaccine (S-FLU) evokes an advantageous memory CD8^+^ T cell immune profile with broad clonal diversity, which can react against viral variants and protect against severe disease without driving the virus to rapidly evolve and escape vaccine-induced immunity.

Although CD8^+^ TCRs are highly specific for their cognate peptide, they can tolerate variations within the CD8^+^ T cell epitope, thus allowing CD8^+^ T cells to respond not only to their cognate peptide but also a range of mutated epitopes (*29*–*31*). TCR diversity within a memory T cell pool is advantageous as it can protect against and limit the emergence of viral CTL escape variants. The value of a broad memory CD8^+^ T cell repertoire is emphasized by the recognition of repertoire diversity maintenance following repeated severe acute respiratory syndrome coronavirus 2 (SARS-CoV-2) infections/vaccinations (*32*) as an important parameter to circumvent the onset of breakthrough variant infections. The mechanisms that enable the maintenance of clonal diversity in the memory T cell pool are unclear. Here, we show that S-FLU immunization, which is inherently non-inflammatory, generates a lung influenza virus NP-specific memory NP-specific CD8^+^ T cell pool with lower affinity and enhanced TCR diversity as compared to influenza virus infection. This resulted in greater capacity of the S-FLU–generated memory NP-specific CD8^+^ T cell pool to respond to viral variants and a reduced ability to drive the emergence of escape variants. While not addressed in this current study, it would be interesting to determine whether affinity and TCR diversity of CD8^+^ memory T cells specific for other influenza virus epitopes are also altered following S-FLU immunization. This finding is in line with earlier reports that also show that S-FLU vaccination can induce cross-reactive subtype immunity, with immunization with the H5 (A/Vietnam/1203/2004) S-FLU vaccine with internal genes from PR8 preventing replication of wild-type (WT) H7N9 (A/Anhui/1/2013) in both mice and ferrets (*13*, *14*).

The tempered inflammatory profile generated following S-FLU immunization influenced the clonotypic diversity of the T cell response as we show that the reintroduction of inflammation by the coadministration of the S-FLU with poly(I:C), an immune stimulating adjuvant, resulted in greater skewing of the TCR repertoire. Malherbe *et al.* (*23*) have previously reported a link between inflammation and the clonotypic diversity of the CD4^+^ T cell response where different types of adjuvants could result in a bias or diversity of clonotypes. In contrast, others have reported the diversity of the CD8^+^ T cell repertoire does not depend on the inflammatory profile. By infecting mice with different viral and bacterial vectors loaded with a fixed epitope, Rudd *et al.* (*33*) investigated the influence of the infectious/inflammatory context on the TCR diversity of the CD8^+^ T cell response and show that although the type of infection in which the epitope was encountered can influence the magnitude of the CD8^+^ T cell responses, TCR β-chain repertoires did not significantly differ among the different infection models. While both these studies focused on the impact of inflammation early during T cell priming, on the circulating memory T cell repertoires, here, we instead explored the impact of the introduction of inflammatory cues later in the response within the tissue compartment and assessed how this affected TCR diversity of tissue-bound memory T cells, a population of immune cells whose functionality (*34*, *35*) and residency (*2*) are known to be heavily influenced by local secondary conditioning events. How the local inflammatory milieu in the lung influences effector CD8^+^ T cell selection and skews the TCR repertoire of the ensuing local memory T cell pool are important questions that will need to be addressed in future studies.

A diverse clonotypic repertoire inversely correlates with viral escape (*36*–*38*). Escape mutants are common for chronic infections including human immunodeficiency virus (HIV) (*39*) and lymphocytic choriomeningitis virus (*40*). While influenza is generally an acute infection, CTL escape mutants are detected in both mouse models and humans (*24*, *28*, *29*, *41*–*43*). Relevant to our presented murine studies, others have shown that CD8^+^ CTL responses directed against influenza virus NP can exert selective pressure on the virus, and variants containing point mutations in the NP_366–374_ epitope readily emerge (*28*). These mutations can both interfere with TCR recognition of the mutant peptide–MHC complex, as well as affecting peptide binding to MHC class I, and collectively affect the ability of cellular immunity to combat infection. We extend these studies by investigating the role of lung Trm to prevent the emergence of viral variants. To achieve this, we transferred lung memory CD8^+^ T cells that comprised Trm of broad clonal diversity into mice devoid of an immune system and showed a reduction in the development of viral escape mutants. While our study does not differentiate the degree of immune pressure exerted by the Trm and the nonresident memory compartment, recent computational studies illustrated that the lung CD8^+^ Trm, and not the circulating memory population, was the predominant selective immune pressure that drove the outgrowth of an NP_366-374_ escape mutant (*44*). Our studies demonstrate the development of viral mutants can be mitigated with a clonally diverse memory T cell pool produced by S-FLU immunization. However, as we and others have shown that lung CD8^+^ Trm in mice undergo rapid attrition (*45*, *46*), future work will need to examine strategies that can extend the durability of broadly diversified lung CD8^+^ Trm evoked by S-FLU vaccination (*47*, *48*).

As multiple CD8^+^ T cell epitopes are recognized simultaneously by CTLs in an immunocompetent host, it is predicated that the overall impact of the loss of recognition of a single T cell epitope on the capacity to control infection is low. Nonetheless, in certain scenarios, viral T cell escape can affect an individual’s capacity to control infection. CD8^+^ T cell responses exert strong selection pressures on HIV, and in patients infected with HIV, T cell escape occurs rapidly. Alarmingly, prophylactic and therapeutic T cell strategies against HIV have shown to result in the strong selection of CTL escape variants (*49*, *50*). Moreover, vaccines that evoke a T cell response against a restricted antigenic target may also be at risk of being outmaneuvered by a rapidly evolving virus, which can profoundly affect the duration of vaccine-induced immunity. Viral genetic variation represents a major strategy exploited by viruses to evade host immune responses, and this represents a major obstacle in the development of T cell–based vaccines and therapies. Vaccines that evoke CD8^+^ T cell memory with diverse TCR repertoires would be greatly beneficial, as this diversity increases cross-reactivity across viral variants and may lessen viral escape.

Our studies highlight that TCR diversity can be built into T cell–based vaccines by manipulating the inflammatory profile evoked by the vaccine formulation. S-FLU vaccination is non-inflammatory, causes minimal lung tissue damage, and generates a clonally diverse cross-reactive lung CD8^+^ memory T cell pool that can protect against severe disease without driving the virus to rapidly evolve and escape. This formulation is an attractive platform to evoke a tissue-bound memory CD8^+^ T cells that are better suited to combat rapidly mutating viruses, including influenza virus, SARS-CoV-2, and HIV.

## METHODS

### Mice and viruses

C57BL/6 (CD45.2), OT-I.CD45.1, RAG-2^−/−^, F5.CD45.1, and F5.CD45.1/2 mice were bred in-house under specific pathogen–free conditions in the animal facility at the Peter Doherty Institute of Infection and Immunity, University of Melbourne, Melbourne, Australia. All experiments were done in accordance with the Institutional Animal Care and Use Committee guidelines of the University of Melbourne. All transgenic strains were maintained on a C57BL/6 background. All mice were female and aged 6 to 8 weeks at the time experiments commenced. Viruses used include X-31 (H3N2, a reassortant virus bearing the HA and neuraminidase genes from A/Aichi/2/1968 and the remaining six internal genes from PR8), PR8 (H1N1 and A/Puerto Rico/8/34), X-31-OVA, or PR8-OVA (which encodes the OVA_257–264_ epitope within the neuraminidase stalk) (*51*) and X-31(DAM) (which carries a mutated “DAM” NP_372-374_ epitope compared to WT “ETM” NP in PR8/X-31) (*20*). For single-cycle influenza virus vaccines including S-FLU(PR8) [S-eGFP/N1(PR8).H1(A/PR/8/1934)], S-FLU(X-31) [S-eGFP/N2(X217)].H3(X-31 A/Aichi/2/1968), or S-FLU(X-31)(DAM); these vaccines are a pseudotyped virus based on the A/PR/8/34 virus genome and modified to have an enhanced green fluorescent protein (eGFP) in place of their own HA, restricting them to a single round of replication (*11*). For total respiratory tract infection, mice were anesthetized with inhalation isoflurane anesthetic and infected in a volume of 30 μl with a sublethal dose of 25 to 50 plaque-forming units (PFU; or as indicated 10^4^ PFU) of PR8 or PR8-OVA; 10^4^ PFU of X-31, X-31-OVA, or X-31-DAM; and 10^6^ TCID_50_ S-FLU(PR8), S-FLU(X-31), or S-FLU(DAM). In some experiments, mice received a secondary “boost” dose of S-FLU 7 days after the primary dose, which was chosen as delaying the time interval between prime and boosting did not affect the size or composition of the memory T cell response. Mice where indicated were also anesthetized with isoflurane and administered 20 μg of poly(I:C) (InvivoGen) or 200 μg of endotoxin-free OVA (Worthington Biochemicals) in a volume of 40 μl. All viruses were sequenced to ensure purity and clonality of the NP_366_ sequence.

### S-FLU viruses

S-FLU viruses that express eGFP in place of endogenous HA and pseudotyped with desired HAs were produced as described (*13*). Genotypes are described using the convention by Powell *et al.* (*11*): The encoded HA and NA replacements are shown between square brackets, followed by HA coating by pseudotyping: [HA replacement/NA origin].HA coat. The S-FLU viruses used in this paper include the following:

1) S-FLU (X-31) = [S-eGFP*/N2(X217)].H3 (X-31). The virus was obtained by reassortment between an S-FLU clone containing six RNA segments from A/PR/8/1934 encoding the internal proteins and the X-217 vaccine strain from NIBSC X-217, which is a 6:2 reassortant between A/PR/8/34 and A/Victoria/361/2011 that contains the HA(H3) and NA(N2) segments from A/Victoria/361/2011 and all internal genes from A/PR/8/34. Reassortant S-FLUs expressing eGFP from the HA expression cassette and N2 derived from X217 were cloned to homogeneity by limiting dilution and propagated in H3 transduced human 2,6-sialtransferase (SIAT-1) cells that provided the pseudotyping H3 from X-31(A/Aichi/2/1968).

2) S-FLU X-31(DAM) = [S-eGFP*/N1(PR8)/NP (E372D,T373A)].H3(X-31). To introduce the epitope recognized by CTL clone F5 (*20*), the cDNA encoding the viral PR8 NP sequence was altered by site-directed mutagenesis in the region of amino acids 366 to 374 from ASNENMETM to ASNENMDAM, and the S-FLU was constructed with this NP sequence. Note that in this virus, the N1 (PR/8) gene is used, not N2. The * over the S-eGFP* signifies high level expression resulting from the modification of an interfering out-of-frame atg codon at position 59 to 62 to aCg in the 3′ untranslated region (3′UTR) (*52*). For S-FLU X-31(DAM), a third out-of-frame atg at 79 to 81 was altered to Tag. S-FLU X-31(DAM) also contains a slightly shorter 5′UTR containing the Eco RI cloning site followed by bases 1511 to 1778 of segment 5 RNA compared to 1275 to 1778 in the original version of S-FLU (*11*).

### Anti-CD8 antibody labeling

Mice were injected intravenously with 3 μg of phycoerythrin (PE)–conjugated antibody to CD8 (clone YTS-169) 5 min before they were sacrificed. Mice were perfused with PBS, and tissues were collected, processed, and stained with allophycocyanin-conjugated antibody to CD8 (anti-CD8; clone 53 -6.7; eBioscience).

### Adoptive transfer and isolation of naïve T cells

Naïve OT-I CD8^+^ T cells isolated from OT-I TCR transgenic mice and naïve F5 CD8^+^ T cells isolated from F5 transgenic mice were purified from single-cell suspensions prepared from the LN and spleen. Cells were purified after a depletion step using antibodies against CD11b (M1/70), F4/80, Ter-119, Gr-1(RB6), major histocompatibility complex class II (M5/114), and CD4 (GK 1.5), followed by incubation with anti-rat immunoglobulin G-coupled magnetic beads (Dynal Biotech) following the manufacturer’s protocols. Naïve OT-I and F5 T cell preparations were 90 to 95% pure as determined by flow cytometry. 10^6^ purified OT-I.CD45.1 or F5.CD45.1 CD8^+^ T cells were labeled with 5 μM CFSE (Sigma-Aldrich) for 10 min/37°C/10% CO_2_ before intravenous injection into mice.

### Flow cytometry

Single-cell suspensions were prepared from spleens and LNs by mechanical disruption. Mice were perfused before the harvest of the lung tissue, which were enzymatically digested for 1 hour at 37°C in 3 ml of collagenase type 3 (3 mg/ml in RPMI 1640 medium supplemented with 2% fetal bovine serum (FBS). Virus-specific CD8^+^ T cells were identified using in-house produced tetrameric complexes of H2D^b^ and the NP_366_ peptide, either the NP(WT) (ASNENMETM) or NP(68)(ASNENMDAM). Cells were incubated with anti-mouse CD16/32 (Fc block, clone 93, BioLegend) for 10 min at room temperature, then stained with PE-conjugated D^b^NP_366_ tetramers for 1 hour at room temperature, washed with EDTA in balanced salt solution with 2% FBS, and then stained with the appropriate mixture of monoclonal antibodies (mAbs) for 30 min on ice. For intracellular cytokine analysis, single-cell suspensions of the lung and spleen were stimulated with the indicated concentration of NP_366_ (ASNENMETM) peptide for 5 hours at 37°C/10% CO_2_ in the presence of GolgiPlug (BD Biosciences) in complete RPMI [10% FBS, 2 mM glutamine, 50 mM 2-β mercaptoethanol (2-ME), penicillin (100 U/ml), and streptomycin (100 μg/ml)] before surface staining for 30 min on ice with the appropriate mixture of mAbs and then intracellularly stained using a Foxp3 fix/perm kit (Thermo Fisher Scientific) according to the manufacturer’s protocol. For peptide titration curves, the %max of IFN-γ^+^ production of either bulk or CD103^+^ memory CD8^+^CD44^+^ T cells stimulated with graded concentrations of NP_366_ peptide was used to calculate the corresponding EC_50_ values. Data were normalized to the proportion of cytokine^+^ cells at saturating concentration (10^−7^ M). A nonlinear regression curve was fitted to calculate the EC_50._ The conjugated mAbs obtained from BD Pharmingen, BioLegend, or eBioscience include mouse: anti-CD8 (53–6.7), anti-CD8 (YTS-169.4), anti-CD3 (17A2), anti–CD45-1 (A20), anti–CD45-2 (104), anti-Va2 (B20.1), anti-Vb11 (KT11), anti-CD44 (1M7), anti-CD103 (2E7), anti-CD69 (H1.2F3), anti–IFN-γ (XMG1.2), anti–TNF-α (MP6-XT22), anti-Ly6G (1A8), anti-CD11b (M1/70), anti–I-A/I-E (M5/114.15.2), anti-CD62L (MEL-14), anti-CD25 (PC61), and anti-KLRG1 (2F1). Vβ-usage analysis was performed using a BD Mouse Vβ TCR Screening Panel Kit (BD Biosciences, San Diego, CA, USA) according to the manufacturer’s instructions. Samples were acquired using a Becton Dickinson LSRFortessa flow cytometer, and data were analyzed using the FlowJo software package (Tree Star Inc., Ashland, OR, USA).

### Flow cell sorting

For NP-specific memory CD8^+^ T cell adoptive transfer experiments, total pulmonary D^b^NP_366_^+^CD8^+^ T cells were sort-purified, and 10^4^ to 2 × 10^4^ cells were intravenously transferred into RAG-2^−/−^ mice. Cell sorting was performed using a BD FACSAria III (BD Biosciences).

### Assessment of cytokine production in BALF

For collection of the BALF, the trachea was exposed and cannulated, and the lungs were lavaged three times with 300 μl of PBS. Cytokine/chemokine concentrations in BALF were measured using LEGENDplex mouse antivirus response standard panel (BioLegend) following the manufacturer’s instructions. Samples were acquired using a Becton Dickinson FACSCanto II flow cytometer, and data were analyzed using the FlowJo software package (Tree Star Inc., Ashland, OR, USA).

### ViroSpot microneutralization assay

Madin-Darby canine kidney (MDCK) cells acquired from the American Type Culture Collection were cultured in Dulbecco’s modified Eagle’s medium (DMEM) supplemented with 10% (v/v) heat-inactivated FBS, 1% penicillin/streptomycin, 1% l-glutamine, and 1% sodium pyruvate. MDCKs (4 × 10^4^ cells per well) in a 96-well flat-bottom plate were incubated with lung tissue homogenates for 1 hour at 37°C/10% CO_2_. A carboxymethylcellulose (CMC) overlay, generated by mixing 1:1 2× DMEM with 6.4% (w/v) CMC salt (Sigma-Aldrich) diluted in distilled water, was then added to restrict viral spread. Plates were incubated overnight at 37°C/10% CO_2_. The overlay was then removed; the cells were fixed in 80% (v/v) of acetone at 4°C, blocked in 5% skim milk powder diluted in 0.05% PBS tween before being stained with mouse anti-influenza A virus NP (CSL Pty Ltd.) followed by secondary anti–mouse-horse radish peroxidase. TrueBlue Peroxidase Substrate (SeraCare) was added, and the wells were imaged using a CTL ImmunoSpot analyzer.

### Next-generation sequencing for influenza virus variants

Total lung RNA was extracted via the QIAGEN RNeasy extraction kit as per the manufacturer’s protocol. The NP gene of influenza A virus sequences were amplified in two fragments with two pairs of A-NP primers using the MyTaq One-Step RT-PCR Kit as described previously (*53*). The two PCR products for each virus were quantified on TapeStation 4200 using D5000 ScreenTape (Agilent), and 100 ng of each PCR product for the same virus was combined and normalized in 30-μl volume for next-generation sequencing (NGS) library construction using Illumina DNA Prep (M) Tagmentation kit (Illumina). IDT for Illumina Nextera DNA Unique Dual Indexes were used for pooling multiple samples together; pooled library was quantified using HSD1000 ScreenTape on TapeStation 4200, then diluted to 150 pM in elution buffer supplied in the library prep kit, loaded to the iSeq100 flow cell, and sequenced on iSeq100 (Illumina). After the NGS run, fastq data were retrieved and analyzed using iterative refinement meta-assembler (IRMA) pipeline (*54*) to generate consensus sequences and variant call format (vcf) files; vcf files were used for mixed base investigation. Primer sequences for A-NP fragment I include A-NP-M13F (TGTAAAACGACGGCCAGTCAGGGTWRATAATCACTCAMTG) and A-NP-M13R (CAGGAAACAGCTATGACCTGRCTCTTGTGWGCTGG). Primer sequences for A-NP fragment II include A-NP-II-M13F (TGTAAAACGACGGCCAGTCTGAGRGGRTCAGTTGC) and A-NP-II-M13R (CAGGAAACAGCTATGACCAGTAGAAACAAGGGTATTTTTC).

### Analysis of the nucleotide diversity at the haplotype and locus level

To measure the diversity of the viral populations, we applied two metrics, i.e., Shannon entropy and nucleotide diversity, at both the locus and haplotype level to the alignment data (in bam file format) generated from IRMA. Specifically, Shannon entropy at locus *l* is calculated as

$$H_{l}=-\sum_{i=1}^{4}p_{i}\log(p_{i})$$

where the sum is calculated over the sum of the four possible nucleotides. The Shannon entropy at locus level for an epitope region (spanning from locus 1 to *L*) can be then calculated by averaging the *H_l_* across all sites

$$H_{\mathrm{locus}}=\sum_{l=1}^{L}H_{l}/L$$

Similarly, the nucleotide diversity at locus level is calculated by computing the mean of *D_l_*, and *D_l_* is the proportion of pairwise differences between alleles at the locus *l*, where *n_i_* copies of the allele *i* are observed under a total sequencing depth of *N*

$$D_{l}=\frac{\sum_{i\neq j}n_{i}n_{j}}{\frac{1}{2}N(N-1)}$$

$$\pi_{\mathrm{locus}}=\sum_{l=1}^{L}D_{l}/L$$

Shannon entropy and nucleotide diversity at haplotype level rely on phasing of the variants at different locus and thus require analyzing aligned NGS reads. We developed a publicly available software epitope_diversity for this particular purpose, details can be found via https://github.com/Koohoko/epitope_diversity. Assuming that there are *M* different haplotypes observed, the Shannon entropy at haplotype level can be calculated as

$$H_{\mathrm{haplotype}}=-\sum_{i=1}^{M}p_{i}\log(p_{i})$$

and nucleotide diversity at haplotype level can be calculated as

$$\pi_{\mathrm{locus}}=\sum_{i=1}^{M}\sum_{j=1}^{M}p_{i}d_{ij}p_{j}$$

where *d_ij_* is the pairwise nucleotide difference between haplotype *i* and *j*. The source code for the computation in this section is available at https://github.com/Leo-Poon-Lab/SFLU_epitope_diveristy (DOI: 10.5281/zenodo.8010071).

### Statistical analysis

Comparison between two study groups was statistically evaluated by unpaired two-tailed *t* test or Mann-Whitney test. Comparisons between more than two groups (single factor) were evaluated using one-way analysis of variance (ANOVA) with Tukey’s multiple comparisons. Two-way ANOVA with Šidák’s multiple comparisons was used to evaluate more than two groups at different time points or cohorts. Comparison between two categorical variables was performed with two-sided Fisher’s exact test. In all tests, statistical significance was quantified as *P < 0.5, **P < 0.01, ***P < 0.001, and ****P < 0.0001. Statistical analysis was performed using GraphPad Prism (v9).
