# Supplementary material for: Single-cycle influenza virus vaccine generates lung CD8+ Trm that cross-react against viral variants and subvert virus escape mutants
Source: Sci Adv. 2023 Sep 8;9(36):eadg3469. doi: 10.1126/sciadv.adg3469 (PMC10491285; doi:10.1126/sciadv.adg3469)
Supplement: Supplementary file 1 — Fig. S1 to S10 [file sciadv.adg3469_sm.pdf]

Science Advances
AAAS

Supplementary Materials for

Single-cycle influenza virus vaccine generates lung CD8$^+$ Trm that cross-react against viral variants and subvert virus escape mutants

Ming Z. M. Zheng *et al.*

Corresponding author: Linda M. Wakim, wakiml@unimelb.edu.au

Sci. Adv. **9**, eadg3469 (2023)
DOI: 10.1126/sciadv.adg3469

This PDF file includes:

Figs. S1 to S10

Supplementary Figures:

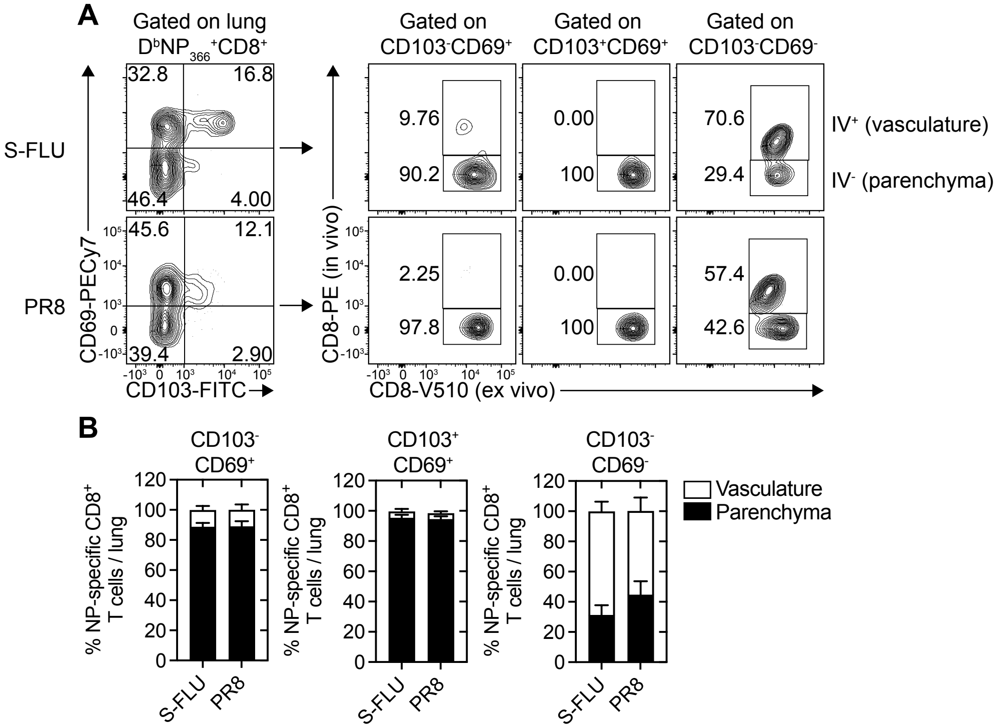

Figure S1. Localization of $D^bNP_{366}^+$ $CD69^+CD103^+CD8^+$ Trm cells in the lung after S-FLU immunisation. B6 mice immunised i.n. with 10^6 $TCID_{50}$ of S-FLU(PR8) or infected with 50 PFU of PR8 received an intravenous injection of anti-CD8-PE ('in vivo') at d30 p.i. and were killed 5 min later for tissue harvest. **(A)** Flow cytometry analysis on the localization of lung $D^bNP_{366}^+CD8^+$ T cell subsets with corresponding **(B)** frequencies for cells that are parenchyma-bound (CD8-IV$^-$) or vasculature-associated (CD8-IV$^+$). Data from 2 independent experiments with n=5-6 mice per cohort. Mean ± SEM.

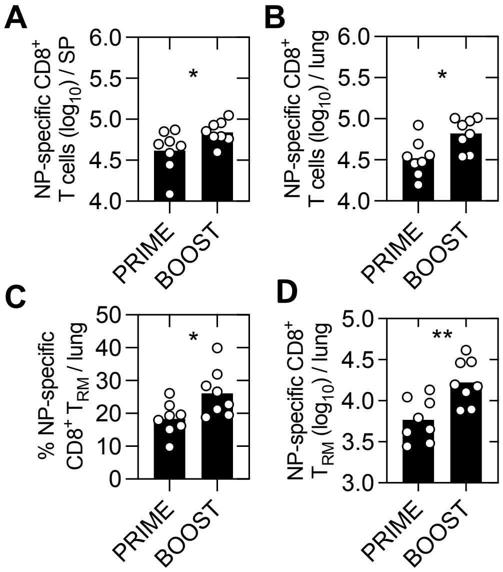

Figure S2. Prime and boost with S-FLU increases the influenza $D^bNP_{366}{}^+$-specific $CD8^+$ T cell response. B6 mice were immunised i.n. with 10^6 $TCID_{50}$ of S-FLU(PR8) (prime) and 7-10 days later given an i.n. homologous boost of S-FLU(PR8/H1N1). Lung and spleen were harvested and analyzed for NP-specific $CD8^+$ T cells 20 days after boost administration. Absolute cell numbers of $D^bNP_{366}{}^+$-specific memory $CD8^+$ T cells in the **(A)** spleen, and **(B)** lung. **(C)** Frequency and **(D)** numbers of $D^bNP_{366}{}^+$-specific $CD69^+CD103^+$ $CD8^+$ lung Trm. Data pooled from 2 independent experiments. Mean ± SEM. Each symbol is a biological replicate. Student t test. **p*<0.05, ***p*<0.01.

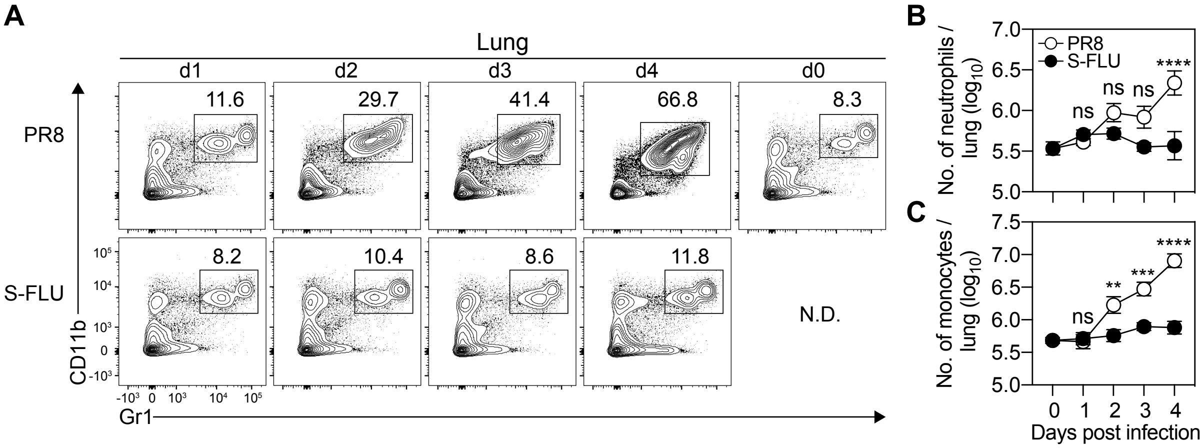

Figure S3. S-FLU immunisation evokes minimal innate immune cell infiltration into the lungs. B6 mice immunised i.n. with 10^6 $TCID_{50}$ of S-FLU or infected with 10^4 PFU of PR8 were analysed for neutrophils and monocytes at d1-4 p.i. in the lung via flow cytometry. **(A)** FACS profiles depicting the proportion of $CD11b^+Gr1^+$ granulocytes in the lungs of S-FLU and PR8 infected mice. **(B-C)** Absolute cell count of **(B)** $Ly6G^+CD11b^+Gr1^+$ neutrophils and **(C)** $Ly6G^-CD11b^+Gr1^+$ monocytes in the lung. Data pooled from 3 independent experiments with n=2-6 mice per timepoint for each cohort. Mean ± SEM. Two-way ANOVA with Šidák's multiple comparison test. *$p<0.05$, **$p<0.01$, ***$p<0.001$, ****$p<0.0001$, ns non-significant.

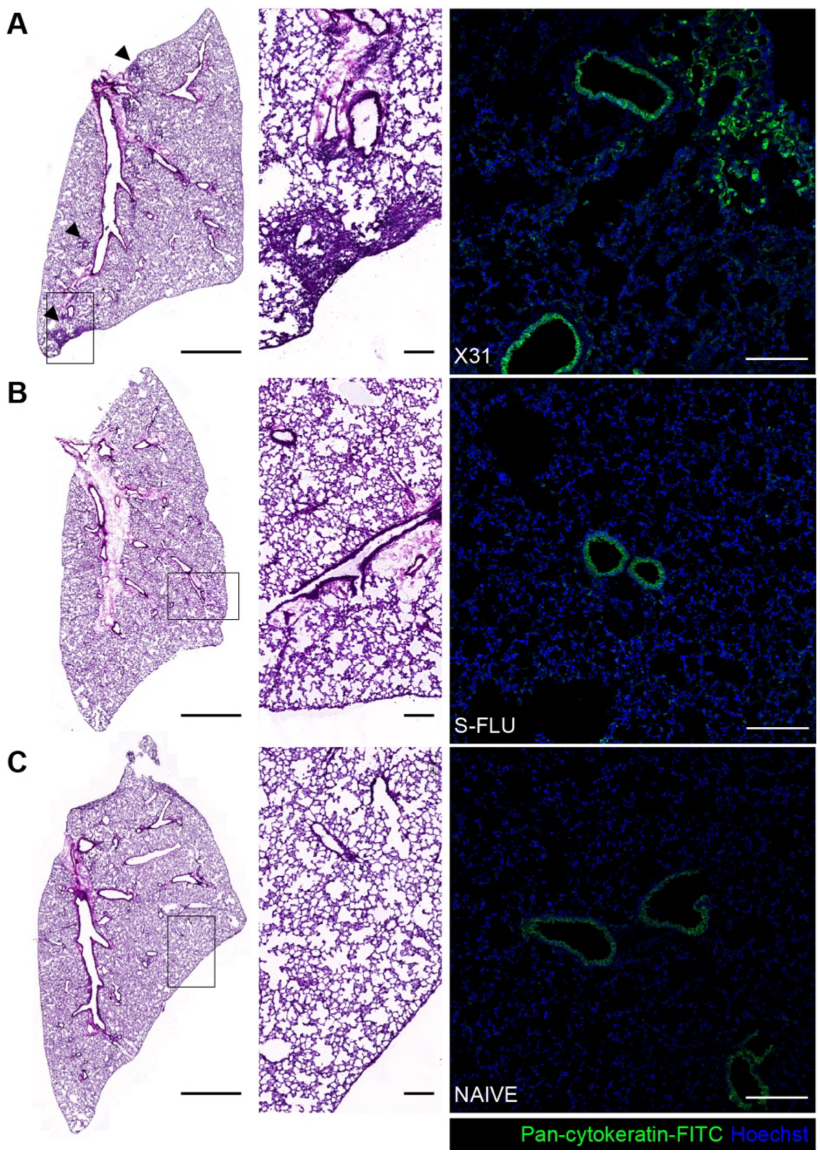

Figure S4. S-FLU immunisation results in minimal lung damage. B6 mice were i.n. administered 10^4 PFU of X-31 or 10^6 $TCID_{50}$ of S-FLU and then 28 days later the lungs were harvested for microscopy. **(A-C)** H&E staining of whole lung (left) with magnified inset (middle) and immunofluorescent imaging of pan-cytokeratin$^+$ aggregates (right) in lungs of **(A)** X-31 infected, **(B)** S-FLU-immunised, and **(C)** naïve mice. Immunofluorescent imaging performed on 40X/1.4 NA objective. Scale bar represents (from left to right) 2000μm, 200μm, and 200μm. Data representative of n=2 mice (n=1 for naïve) from 1 independent experiment.

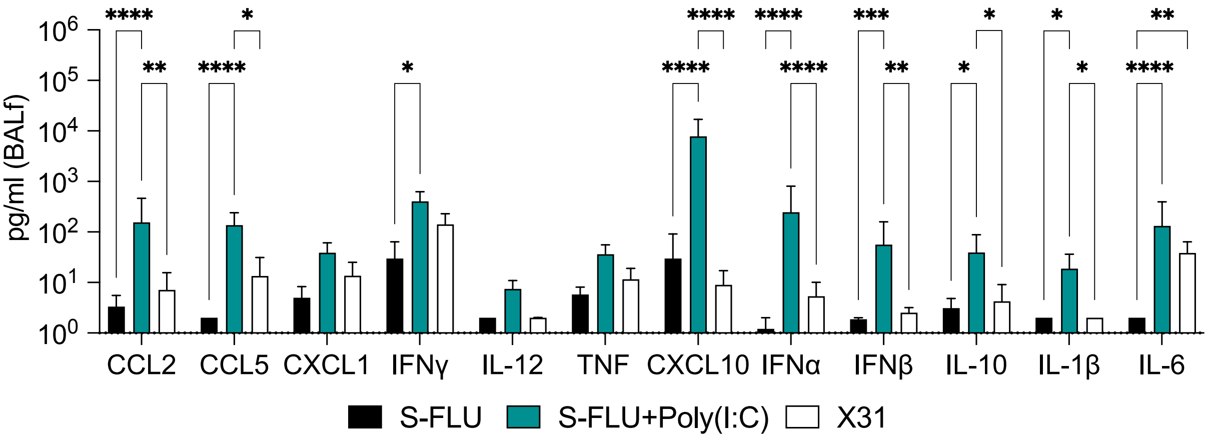

Figure S5. Secondary boosting of S-FLU-immunised mice with poly(I:C) adjuvant increases the local inflammatory milieu. B6 mice were i.n. infected with 10^4 PFU of X-31, i.n. immunised with 10^6 $TCID_{50}$ of S-FLU or i.n. administered 20µg of poly(I:C) at d7 p.i. of S-FLU immunisation. At d8 p.i., the concentrations of cytokines and chemokines in the BALF was measured by cytometric bead array. Data pooled from 2 independent experiments with n=5 mice per cohort. Mean ± SEM. Two-way ANOVA with Šidák's multiple comparison test. **p*<0.05, ***p*<0.01, ****p*<0.001, *****p*<0.0001, ns non-significant.

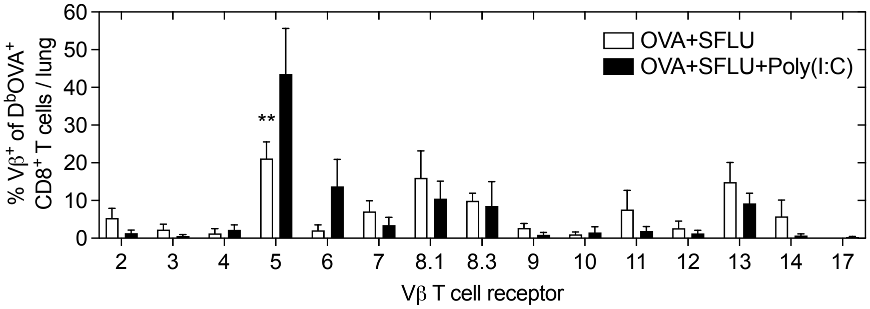

Figure S6. The skewing of the Vβ T cell receptor repertoire following the addition of an inflammatory adjuvant is unrelated to antigen dose. B6 mice were i.n. administered with OVA (200μg) and S-FLU(X-31) (10^6 $TCID_{50}$), and 7 days later received Poly(I:C) (20μg) i.n. or remained untreated. At d21 p.i., the Vβ T cell receptor repertoire was analysed by flow cytometry. Data are presented as mean ± SEM of normalised frequencies of individual Vβ T cell receptors of $D^bOVA^+CD8^+$ T cells in the lung. Data are compiled from 2 independent experiments with n=4 mice per group. Two-way ANOVA with Šidák's multiple comparison test. $^{**}p<0.01$.

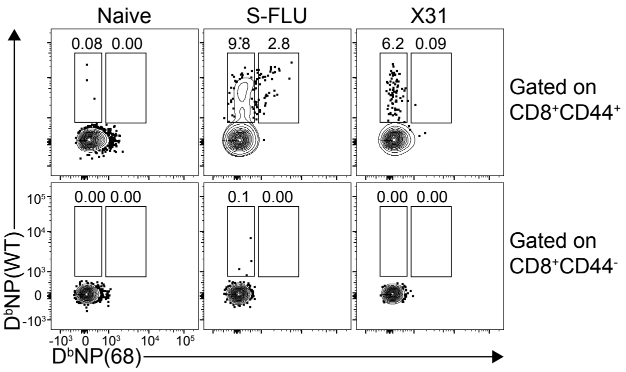

Figure S7. Cross-reactive NP-specific CD8$^+$ T cells generated by S-FLU immunisation are restricted to the antigen-experienced CD8$^+$CD44$^+$ T cell subset. B6 mice i.n. administered with 10^6 $TCID_{50}$ of S-FLU(X-31) or 10^4 PFU of X-31 were analysed for cross-reactive NP-specific CD8$^+$ T cells at d28 p.i. via flow cytometry. FACS profiles depicting the proportion of cross-reactive memory D^{b}NP(WT)$^+$D^{b}NP(68)$^+$ CD8$^+$ T cells gated on CD8$^+$CD44$^+$ and CD8$^+$CD44$^-$ subsets in the lung. Data representative of n=7 mice (n=1 naïve) per cohort from 2 independent experiments.

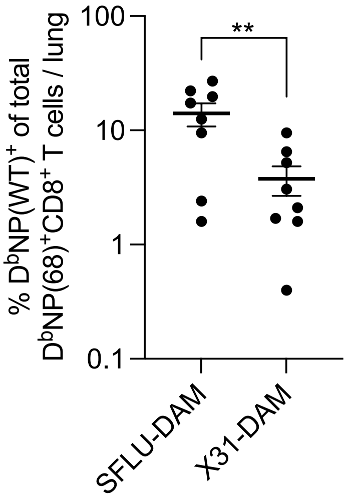

Figure S8. S-FLU(DAM) immunisation generates cross-reactive NP(WT)-specific CD8$^+$ T cells. Frequencies of cross-reactive D^{b}NP(WT)$^+$CD8$^+$ T cells in the total D^{b}NP(68)$^+$CD8$^+$ T cell population in the lungs of B6 mice i.n. immunised with 10^6 TCID$_{50}$ of X-31 S-FLU(DAM) or infected with 10^4 PFU of X-31(DAM) at d21 p.i. as measured by flow cytometry. Data pooled from 2 independent experiments with n=8 mice per cohort, presented as mean ± SEM. Symbols indicate individual mice. Two-tailed unpaired t test. **p<0.01

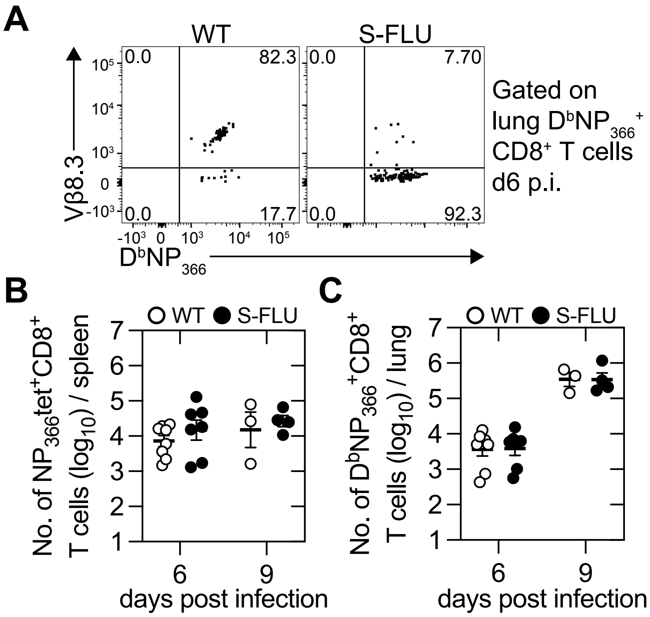

Figure S9. Lung homing capacity of NP-specific $CD8^+$ memory T cells recovered from the lung of S-FLU and WT influenza virus infected mice. Memory $D^bNP_{366\text{-}374}{}^+$ $CD8^+$ T cells from WT influenza virus or S-FLU-administered B6 mice were i.v. transferred into $RAG^{-/-}$ mice, rested, and then infected with 10^4 PFU of X-31 i.n. **(A)** FACS profiles gated on $CD8^+$ T cells in the lung on day 6 p.i. depicting WT and S-FLU $D^bNP_{366}{}^+$ $CD8^+$ T cells expressing the Vβ8.3 T cell receptor. The number of $D^bNP_{366}{}^+$ $CD8^+$ T cells in the **(B)** spleen and **(C)** lung at d6 and 9 post infection. Data pooled from 3 independent experiments with n=3-7 mice per cohort, presented as mean ± SEM. Symbols indicate individual mice.

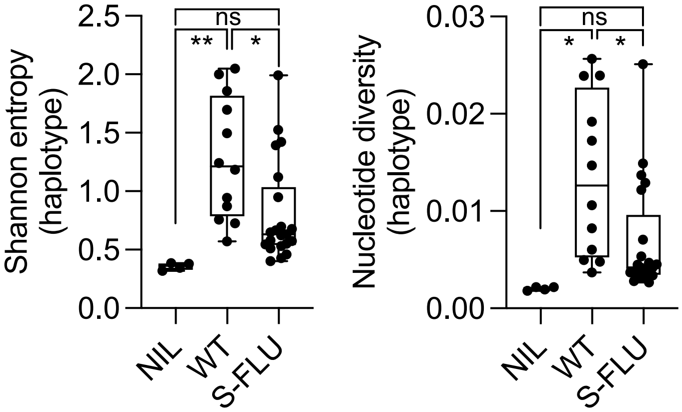

Figure S10. S-FLU-generated lung memory NP-specific CD8$^+$ T cells drive reduced influenza virus epitope diversity. Pulmonary memory $D^bNP_{366\text{-}374}^+$ CD8$^+$ T cells from WT influenza virus or S-FLU-administered B6 mice were i.v. transferred into RAG$^{-/-}$ mice or were not transferred (NIL), rested, and then infected with 10^4 PFU of X-31 i.n. Shannon entropy and nucleotide diversity analysis on the haplotypes of the viral populations detected in the lungs of RAG$^{-/-}$ mice. Data pooled from 4 independent experiments with n=4-21 mice per group. Symbols indicate individual mice. Box plot lines display median and the first/third quartile. One-way ANOVA with Tukey's multiple comparison test. *p<0.05, **p<0.01, ns non-significant.
